# Artificial intelligence predicts normal summer monsoon rainfall for India in 2023

**DOI:** 10.1038/s41598-023-44284-3

**Published:** 2024-01-17

**Authors:** Udit Narang, Kushal Juneja, Pankaj Upadhyaya, Popat Salunke, Tanmoy Chakraborty, Swadhin Kumar Behera, Saroj Kanta Mishra, Akhil Dev Suresh

**Affiliations:** 1 Department of Computer Science and Engineering, Indraprastha Institute of Information Technology Delhi, Delhi, India; 2 Centre for Atmospheric Sciences, Indian Institute of Technology Delhi, Delhi, India; 3 Center for Global Change Science, Massachusetts Institute of Technology, Cambridge, Massachusetts USA; 4 Department of Electrical Engineering, Indian Institute of Technology Delhi, Delhi, India; 5 Application Laboratory, Japan Agency for Marine-Earth Science and Technology, Yokohama, Japan; 6 Department of Physics, Indian Institute of Science Education and Research Tirupati, Tirupati, India

**Keywords:** Environmental impact, Atmospheric science, Energy and society

## Abstract

Inaccuracy in the All Indian Summer Monsoon Rainfall (AISMR) forecast has major repercussions for India's economy and people's daily lives. Improving the accuracy of AISMR forecasts remains a challenge. An attempt is made here to address this problem by taking advantage of recent advances in machine learning techniques. The data-driven models trained with historical AISMR data, the Niño3.4 index, and categorical Indian Ocean Dipole values outperform the traditional physical models, and the best-performing model predicts that the 2023 AISMR will be roughly 790 mm, which is typical of a normal monsoon year.

## Introduction

An accurate forecast of All Indian Summer Monsoon Rainfall (AISMR) is crucial for policy and decision-making, with applications in a broad range of socioeconomic sectors, including agriculture, energy, water resources, health, and disaster management. Given that a significant portion of the population relies on agriculture, an accurate forecast of AISMR is necessary to ensure sufficient water availability for crops, a fair allocation of water resources, and prevent price inflation due to a scarcity of essential goods. Recent history provides several examples of unanticipated severe droughts that unfolded during the years 2002 and 2004 against the predicted normal monsoon rains^1^. These events resulted in severe socio-economic repercussions, highlighting the importance of reliable seasonal AISMR forecasts for policy decisions.

Conventionally, physical models have been utilized to create seasonal forecasts, which offer a probabilistic prediction along with an ensemble spread that indicates the level of uncertainty associated with the event^2–4^. However, these models have several limitations such as, being computationally expensive, having a high sensitivity to parameter initialization, and having limited flexibility in input parameters^4,5^. Additionally, the forecast skill of these models relies on their ability to simulate the relationship between predictors and predictands, which is influenced by factors like model fidelity, boundary conditions, and the ability to simulate ocean-atmospheric coupling and feedback processes^6,7^. Typically, agencies engaged in monsoon forecasting normally use the multi-model ensemble mean from these physical-based climate models but recent decades have seen a drop in forecast skill across many models^8,9^. Analyzing the seasonal forecasts made by these physical models for 2002–2022, we found that only six out of 21 years were within the specified range (https://mausam.imd.gov.in/imd_latest/contents/seasonal_forecast.php). The challenges associated with improving seasonal forecasting with the physical models include realistic initialization of initial conditions, accurate representation of climate dynamics, including seasonal variability, and sufficiently sampling the forecast spread arising from uncertainties in the initial conditions. Addressing these challenges is difficult given the considerable cost of initializing the initial state, the increasing complexity of the models, and the finite number of observations for forecast verification^10^.

Recently artificial intelligence (AI) and machine learning (ML)^11^ have emerged as powerful and promising techniques for predicting ocean and atmospheric variations. These techniques carry enormous potential because of their capability to dig through the large volume of observational and model data and find emerging patterns much more efficiently than conventional physical models. Many forecasting agencies like the National Oceanic and Atmospheric Administration (NOAA)^12^, the Indian Meteorological Department (IMD) (https://moes.gov.in/schemes/atmospheric-climate-science-and-services?language_content_entity=en), and the UK Met Office^13^ are now trying to develop forecast systems by using these AI and ML techniques together with the physical models for forecasting important climate parameters such as rainfall and temperature. With the advancements in the field of AI, different techniques in machine learning, deep learning, computer vision, and big data analytics have been employed in solving problems related to climate sciences like multi-year ENSO forecast^14^, multi-seasonal IOD forecast^15^, hurricane detection, wildfire detection, forecasting rainfall^16,17^. Data-driven methods, including machine learning models such as Linear Regression, Support Vector Regression (SVR), XGBoost, and Random Forests, as well as neural models such as Convolutional Network Networks (CNNs), Recurrent Neural Networks (RNNs), and Transformers, have been employed in many studies for rainfall forecasting. These models are particularly preferred due to their better forecast ability when compared to physical models, their flexibility in input parameters, and their ability to capture non-linear and complex relationships^3,5,16–20^.

The predictability in seasonal forecasts is largely derived from the slowly varying coupled dynamics of the land, atmosphere, and ocean, which include predictable variations in soil moisture, sea ice, snow cover, and sea surface temperature (SST)^7,21,22^. Particularly, the slowly varying ocean-atmosphere conditions associated with tropical climate phenomena El Niño-Southern Oscillation (ENSO) and the Indian Ocean Dipole (IOD) play a crucial role in interannual variability of Indian summer monsoon rainfall^8,23^. As a result, they are widely considered as the major sources of predictability for the seasonal forecast^8^. Therefore, by leveraging advanced AI and ML techniques to account for these sources of predictability, it may be possible to develop a more reliable and actionable seasonal forecast for AISMR^21,22^.

Here we present such a data-driven approach by taking into account the primary drivers of interannual variability, ENSO and IOD, and by employing several empirical models to predict the AISMR of 2023. The results obtained using Long Short-Term Memory (LSTM), an RNN model that outperforms all other empirical models in the training period, indicate that in 2023, India will receive ~ 790 mm of rainfall, which falls in the normal range of the tercile category (see Methods).

## Results

### ENSO and IOD as predictability sources

**Figure 1:**
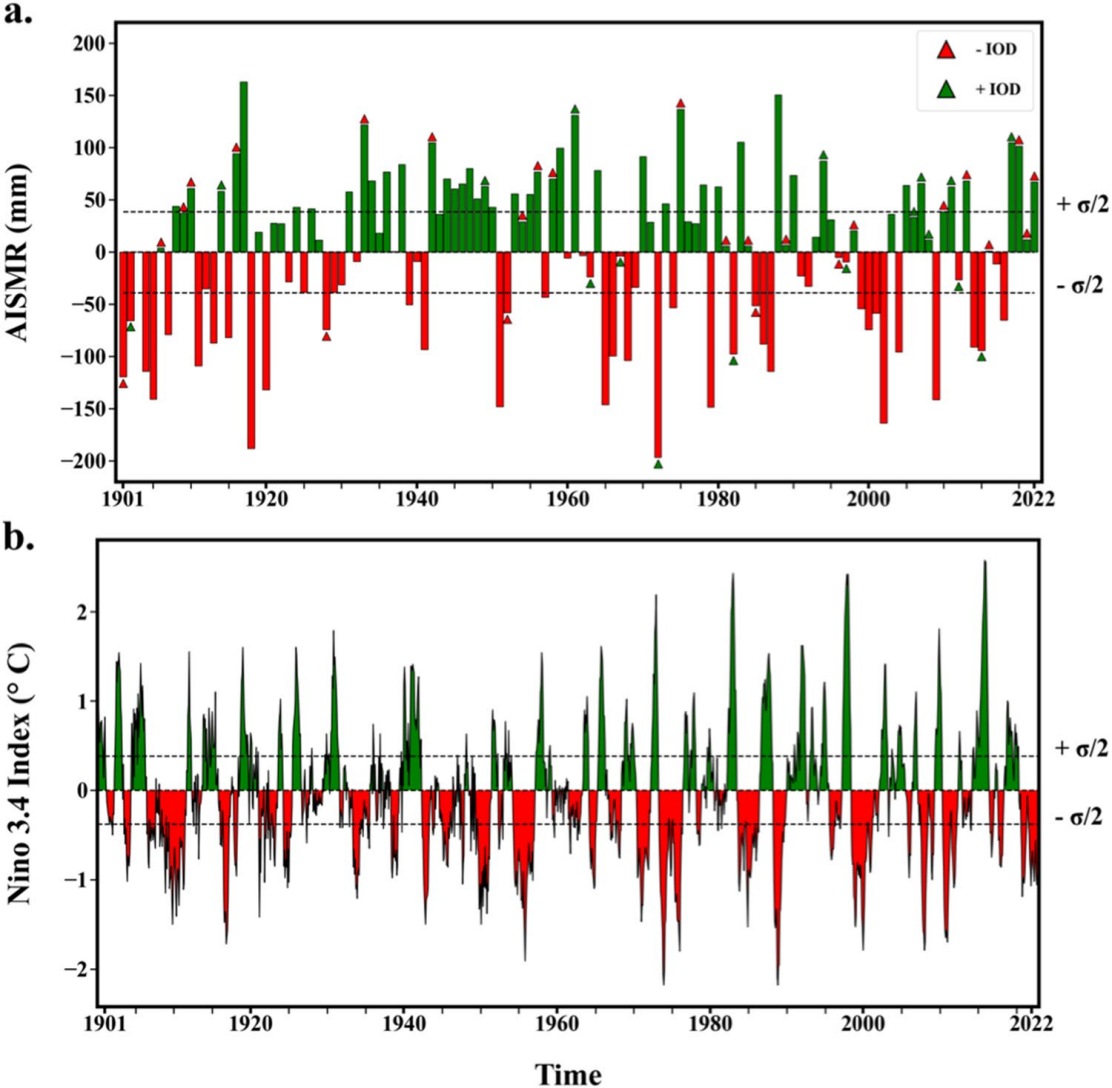
Time evolution of AISMR, IOD events, and Niño3.4 SST anomaly. (**a**) The anomaly of AISMR from the long-term mean for the Indian Summer Monsoon months (June to September) over the period 1901 to 2022 and the IOD event for the corresponding year. (**b**) The evolution of monthly Niño3.4 Index over the same period.

The interannual variabilities of AISMR are mainly contributed by ENSO and IOD either individually or as a combined effect of the two^24,25^. Due to the slow evolution of these drivers as coupled ocean-atmospheric phenomena in the tropics, early appearance of their signals in tropical SST anomalies far in advance of the monsoon season offers AISMR a reliable window of predictability with a significant lead time. Figure 1a shows the interannual variation of AISMR with anomalies indicating both above and below the long-term mean (858.02 mm) from 1901 to 2022, along with the IOD events represented by overlaid markers. The IOD is characterized by the difference in the sea surface temperatures between the western and eastern tropical Indian Oceans^26^, with positive and negative IODs associated with a respective increase and decrease in rainfall in India. However, the relationship between the two is not so straight forward as evident from Fig. 1a and that uncertainty in a source of AISMR predictability poses a challenge in its forecast. Besides IOD, the ENSO mode characterized by anomalous changes in SST and associated circulations in the equatorial Pacific is traditionally linked to monsoon variability. Indeed, it is clear from the time evolution of the Niño3.4 index shown in Fig. 1, which includes both El Niño and La Niña (positive/negative ENSO) events, that there is a strong relationship between ENSO and AISMR. The occurrence of El Niño events tends to reduce the rainfall over the Indian subcontinent resulting in a deficit of rainfall (for instance, in 1904, 1972, and 1987) and vice versa (for instance, in 1910, 1933, and 1988). While the individual effects of these drivers (such as IOD and ENSO) on AISMR can be anticipated to some extent, their combined non-linear effect^15,27^ and the significant sensitivity of AISMR to the magnitude and specific types of these modes^28^ of variability can lead to uncertainties in forecasting AISMR. This, in turn, can result in inaccurate predictions. For example, in 1997, the positive phase of IOD coincided with a strong El Niño event, yet India recorded near-normal rainfall, highlighting the difficulty in accurately forecasting AISMR in the presence of complex interactions between ENSO and IOD^28^.

### Development of a skillful data driven model

**Figure 2:**
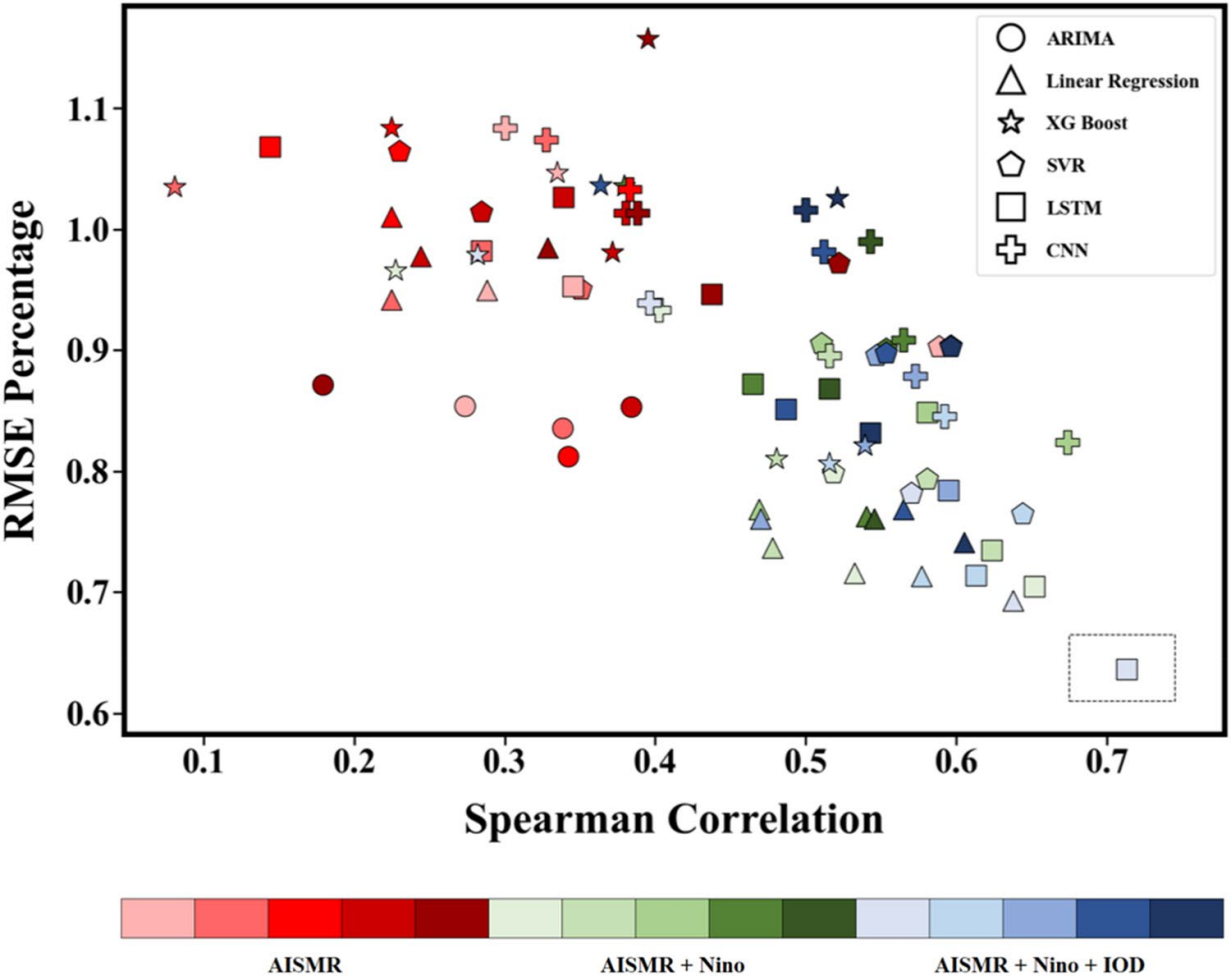
Skill of different data-driven models in the test period. The RMSE percentage vs. temporal correlation (Spearman) for AISMR using multiple data-driven models depicted by the markers on the test period from 2002 to 2022 for three different suites of experiments—AISMR, AISMR + Niño, and AISMR + Niño + IOD (for further details, refer to Methods). The RMSE percentage and Spearman correlation are calculated in reference to the observed AISMR values from IMD. The color shades represent different lookback periods, i.e., the number of years under consideration for the AISMR data to train the models, that range from 5 to 25 years for each suite of experiments.

Incorporating the two major drivers of AISMR, namely IOD and ENSO, we present a data-driven approach in an attempt to provide a reliable seasonal forecast of AISMR, an alternative to forecasts using conventional physical models. Here, a suite of successive experiments of AISMR forecast has been carried out employing multiple statistical machine learning, and deep learning models using three different datasets – (a) only historical AISMR (AISMR, hereafter), (b) historical AISMR and Niño3.4 index (AISMRNiño, hereafter), and (c) historical AISMR, Niño3.4 index, and categorical IOD data (AISMRNiñoIOD, hereafter) for a range of lookback windows. The lookback window denotes the period of the historical AISMR data considered for predicting the oncoming AISMR (see Methods for details). Figure 2 shows the model performance in each set of experiments validated using RMSE percentage and Spearman correlation for the test period, i.e., 2002 to 2022, by comparing model predictions and observed AISMR values from IMD.

We apply statistical machine learning models, namely linear regression, SVR, XGBoost, ARIMA, SARIMA, and deep learning models, namely LSTM and CNN. Our findings indicate that the models trained only on the AISMR dataset (see methods) underperform with a higher RMSE percentage (0.8 to 1.1) and lower spearman correlation (0.2 to 0.4) in comparison to those trained on the AISMRNiño dataset (with a lower RMSE percentage and an improvement in the spearman correlation up to 0.67). A further improvement was observed using the AISMRNiñoIOD dataset which produced the best results with 0.64 RMSE percentage and spearman correlation of 0.71. Moreover, the statistical significance of the correlations is evaluated using the mean of the *p*-values and its standard deviation for each dataset, and the successive improvement in the *p*-values from AISMR dataset (*p*-value = 0.2) to AISMRNiñoIOD (*p*-value = 0.0247) is noteworthy (Supplementary Table. 1). The results suggest that a 5-year lookback period yields superior outcomes for most of the models when compared to other lookback periods. This may be attributed to the dataset's small size, as increasing the number of features with a small dataset often leads to a decline in model performance. Furthermore, of the various models trained, LSTM produces the best results, with the highest Spearman correlation and the lowest RMSE percentage. This can be attributed to LSTM's ability to capture the most pertinent characteristics of a series and selectively disregard unimportant parts. Incorporating information related to the drivers like ENSO and IOD to the AISMR dataset has helped in improving the performance of the models, indicating that data-driven models are able to capture the non-linear interaction of different drivers and their combined teleconnections. Based on this skill assessment, LSTM model trained on the AISMRNiñoIOD dataset with a lookback period of five years size has been used to predict the AISMR for the year 2023 (Fig. 2).

### Forecast for the year 2023

**Figure 3:**
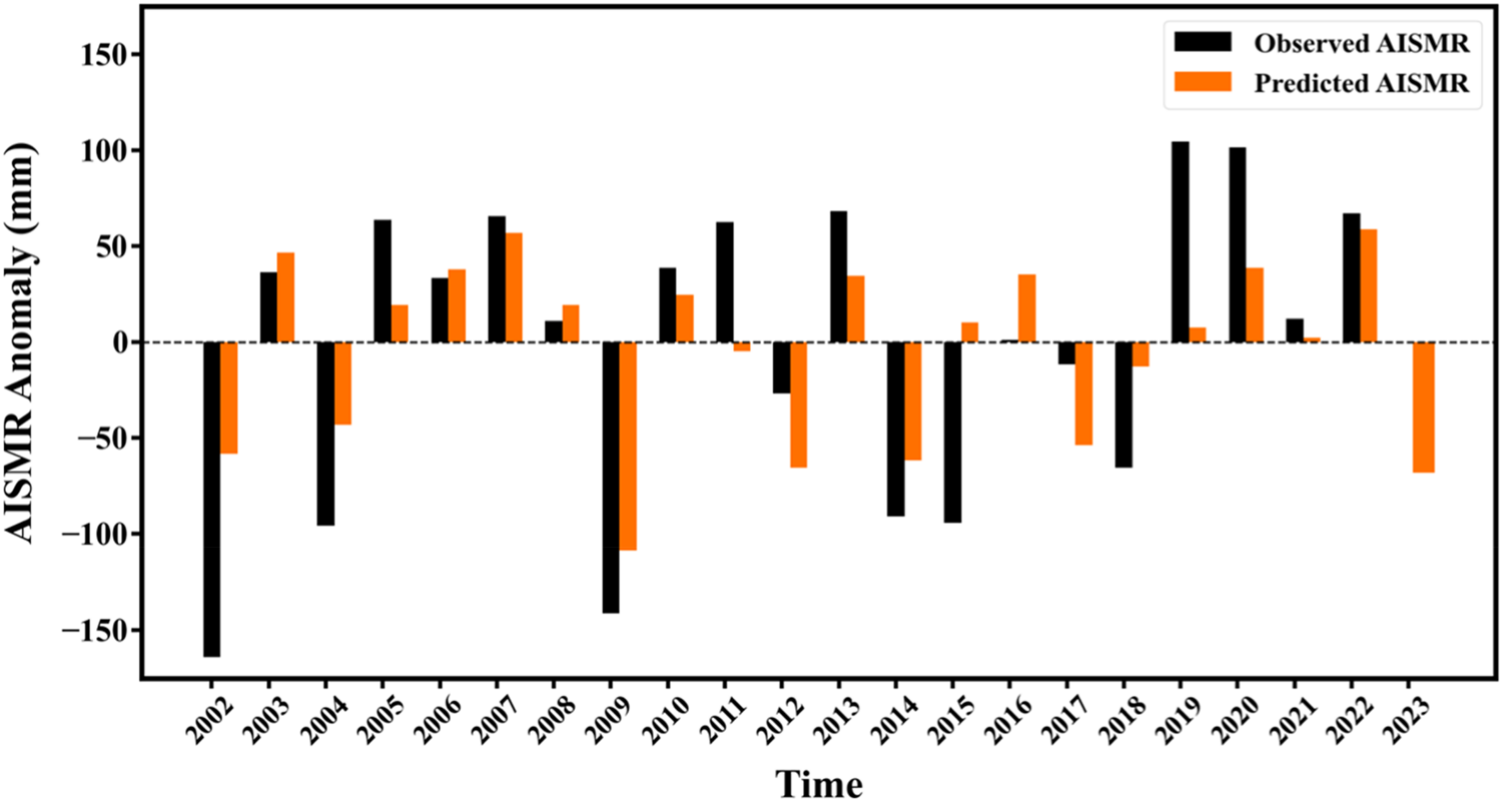
AISMR Forecast for the year 2023 using the best-performing model. The observed AISMR anomaly from IMD, along with the predicted AISMR anomaly using the best-performing model (LSTM-lookback 5) for the test period from 2002 to 2022. The same is extended to include the forecast for the year 2023.

Figure 3 shows the observed AISMR values as provided by IMD (https://mausam.imd.gov.in/imd_latest/contents/seasonal_forecast.php#), along with the predicted values using our best-performing model, i.e., LSTM during the test period (2002–2022). Our analysis reveals that the LSTM model demonstrates a successful forecast rate of approximately 61.9% during the evaluated period, which is significantly higher than the success rate of the physical models utilized by IMD, which is approximately 28.57%. Specifically, the actual  ± 5% range of seasonal cumulative rainfall variations is predicted 13 times by LSTM model, as opposed to 6 times predicted by IMD in their pre-monsoon forecast. This significant improvement in success rate, achieved through the use of a data-driven model, is a promising development that could support the transition of rainfall forecast systems from physical models to more innovative models. Additionally, we have found that the LSTM model exhibits even greater skill in predicting the sign of annual precipitation variation with reference to the long-term mean. Specifically, the predicted values align with the actual value for 19 of 21 years. This highlights the model’s ability to replicate the long-term patterns of precipitation, which is a crucial component of accurate forecasting of different categories of AISMR. The only two years that LSTM fails to categorize the seasonal rainfall are 2011 and 2015. Further, using the same model and approach, the seasonal rainfall forecast for the year 2023 is generated. The forecast took into account the variations in Niño3.4 and DMI indices at different lead time, which suggested to be an El Niño and positive IOD year (see Methods).

The LSTM model predicts that India will experience normal rainfall of approximately 790 mm in 2023. It should be noted that this predicted rainfall is close to the threshold of the normal and below normal categories and hence, cautious measures should be adopted to prevent any adverse effects of a below normal monsoon season. The co-occurrence of El Niño and IOD seem to offset the individual effects and result in a normal rainfall over the oncoming season through atmospheric passage as was observed in the great El Niño year 1997^28^. Our findings suggest that the inter-basin linkage between the Pacific and Indian Oceans plays a crucial role in predicting seasonal AISMR, thereby highlighting the significance of this connection as a reservoir of predictability. This is consistent with the ongoing discussions in the scientific community that emphasize the importance of the complex interactions between the two ocean basins and their impact on weather and climate patterns^27,29^.

## Conclusion

Our study demonstrates the significant forecast skill exhibited by data-driven models for predicting seasonal AISMR. If these data-driven techniques can tap the changing nature of teleconnections and the non-linear interactions in a warming climate that the physical models are often unable to capture, it will considerably enhance the skill in the seasonal forecast of AISMR. We also believe that findings in our study could encourage further efforts towards building a new generation of seasonal forecast systems by integrating physical and data-driven models so as to as complement each other and provide more reliable and accurate seasonal forecasts. A "normal" monsoon rainfall season, as forecasted by our data-driven model, for the year 2023 is a good sign for the country's overall economic condition and welfare since a significant portion of the population is dependent on agriculture. Upon comparing our predicted value with that of the actual observed accumulated AISMR value (up to September 18, 2023) obtained from the IMD (https://mausam.imd.gov.in/responsive/rainfallinformation_msd.php?msg=C, accessed on 19^th^ September, 2023), we find that the difference between the predicted and the observed AISMR is less than 5%, which is reasonably acceptable. The values of the actual accumulated AISMR can be safely assumed to remain unchanged since the monsoon has already started to recede by the time of reporting. It is also noteworthy that the prediction was made in the month of January, providing a lead time of four months. Although the forecast may not be useful for farmers at a granular scale, however, it would be useful in seasonal scale planning of water resource and insurance. It would also facilitate other stakeholders with an adequate time window to anticipate and optimize the necessary resources.

Besides agriculture, a growing demand for seasonal forecasts in a multitude of applications in energy, water management, risk assessment, and disaster mitigation has instigated efforts in search of alternative prediction approaches in recent years. In the current age of data science, the potential for enhancing prediction skills using AI and ML is abundant. However, several challenges must be addressed. For instance, the data in climate science is limited for training data-driven models. Also, a lack of interpretability poses significant obstacles. Nevertheless, additional sources of predictability that have emerged in recent times, such as Atlantic influence on AISMR at sub-seasonal to seasonal scale^30,31^, new modes of principal components of AISMR^9^, and unexplored influence of stratosphere-troposphere coupling on monsoon surface climate^22^ may provide an extended window for monsoon predictability.

On another note, the point forecast of AISMR averaged for spatially diverse country like India may not be sufficient for guiding effective action plans. Therefore, similar data-driven approaches can be extended to cover a wide range of homogenous regions of the Indian subcontinent at a spatial resolution of district-to-sub-district scales. This study is an attempt to predict the AISMR for the oncoming summer season by exploiting the emerging cutting-edge tools, but it can only be validated as and when the monsoon ends, and we obtain a definite AISMR value. Even if the forecast misses to capture the actual observation this time, which is very unlikely based on the results from the testing period, the exercise remains valuable as an exploratory endeavor. Through the use of data-driven models, the exercise highlights the potential for enhanced extended range forecasts in the future, even in cases where categorical identification of AISMR rainfall is not possible.

## Methods

### Model training data

The experiments conducted in this study use AISMR, Niño3.4 index, and Indian Ocean Dipole (IOD) data for training the data-driven models (statistical machine learning and deep learning models). The all-India seasonal mean of the cumulative daily rainfall gridded data from IMD for June to September for the period 1901–2022 is used to obtain the yearly AISMR values. The AISMR data was pre-processed by discarding the erroneous values of rainfall (less than 0 mm). The ENSO events are characterized using the monthly Niño3.4 index, provided by NOAA^32^, which measures the sea surface temperature anomaly in the equatorial Pacific Ocean (5S-5N and 170W–120W). In addition, the categorical IOD data representing positive IOD, negative IOD, or neutral IOD years provided by Japan Meteorological Agency (https://ds.data.jma.go.jp/tcc/tcc/products/elnino/iodevents.html) were used to feed the models. Owing to the categorical nature of the data, one-hot encoding of the IOD data is done before training the models. Figure 1a shows the interannual variation of AISMR anomaly about the long-term mean value from 1901 to 2022, along with the IOD events represented by overlaid markers, Fig. 1b shows the monthly variation of Niño3.4 index anomaly from 1901 to 2022.

### Categorizing IOD events

Dipole Mode Index (DMI) is used as a quantitative measure for characterizing IOD which is defined as the difference between the SST anomalies between tropical western (50°E to 70°E and 10°S to 10°N) and eastern (90°E to 110°E and 10°S to 0°S) India Ocean. IOD events are categorized into three categories: Positive, Negative, and Neutral. Positive IOD events are identified when the three-month running mean dipole mode index (DMI) is  + 0.4 °C or above for at least three consecutive months between June and November. Negative IOD events are identified when the three-month running DMI is  − 0.4 °C or below for at least three consecutive months between June and November.

### Tercile category

The seasonal forecast is classified into three categories: below normal (< 90% of the Long Period Average (LPA)), normal (90–110% of the LPA), and above normal (> 110% of the LPA). The LPA of observed rainfall data from IMD for the period 1901 to 2001 is found to be 858.02 mm.

### Statistical machine learning, and deep learning models

In our experiments, a wide range of models was trained – statistical machine learning models, namely Linear Regression, Support Vector Regression (SVR), XGBoost, ARIMA and SARIMA; deep learning models, namely Long Short-Term Memory (LSTM), and CNNs. Here we briefly describe these models. Linear Regression assumes a linear relationship between the input variables and the target variable. It aims at finding a line that best describes the relationship between the input and target variables by gradient descent optimization. SVR is a type of Support Vector Machine (SVM) that is used for regression analysis. SVR uses different types of kernel functions such as linear, polynomial, radial basis function (RBF), and sigmoid to transform the data into higher dimensions, therefore making the data, which is linearly inseparable in the lower dimensions, separable in the higher dimensions. The experiments were conducted using all four types of kernel functions, and the best results were obtained using the RBF kernel. Extreme Gradient Boosting (XGBoost) is an efficient implementation of the gradient boosting algorithm, which is capable of finding a non-linear relationship between the input and the target features^33^. Gradient boosting is an ensemble-based algorithm that capitalizes several weak models and combines them to form a strong model^34^. XGBoost has a large number of parameters that need to be specified before training the models, like the maximum depth of the tree, the learning rate, and the number of decision stumps used for the ensemble. In the experiments, we used learning rate = 0.3, max_depth = 6, and 100 decision stumps for training the models. Autoregressive Integrated Moving Average (ARIMA) model is a statistical model that takes three hyperparameters (*p*, *d*, *q*) into account where *p* is the order of the autoregressive model, *d* is the degree of differencing required to make the time-series stationary, and *q* is the order of the moving-average. ARIMA models require only the historical data of the time series to make the forecast and assume that the time series becomes stationary after the differencing operation. Seasonal-ARIMA (SARIMA) is an extension of ARIMA that takes into account the seasonal component of the time series, which the ARIMA model does not. Both ARIMA and SARIMA are primarily used for time series analysis. Fine-tuning for ARIMA and SARIMA was done using the grid search method, where different combinations of (*p, d, q*) triplets were tried for model training, and the triplets (15, 0, 3) and (20, 0, 3) were giving good results. Time series analysis using deep learning has also gained momentum in the past few years, with LSTM being the most commonly used among them. LSTM is a Recurrent Neural Network with the ability to selectively forget information that is not relevant and only persist in the important parts of the sequence. LSTMs are capable of learning patterns from any type of sequential data, and they do not rely on the stationarity assumption of the time series. Fine-tuning of the LSTM model gave the best results for batch size = 20 for 125 epochs, and to reduce the overfitting, dropouts were used. The Adam optimizer was used with a mean squared error loss for training the model. Convolutional Neural Network (CNN) is a feed-forward neural network that uses a convolutional layer to capture the spatial and temporal dependencies in the data using convolution^35^. In the experiments, for each 1-D convolution layer and feed-forward layer, RELU activation is used. Similar to LSTM, the Adam optimizer and mean squared error loss are used for model training.

### Experimental setup and testing

Of the total 122 years of historical data from 1901 to 2022, the latest 21 years, i.e., from 2002 to 2022, are used for testing the data-driven models, and the remaining 101 years, from 1901 to 2001, were used for model training. Based on the dataset used for training the models, the experiments can be classified into three categories. The primary goal for each category of experiments is to predict AISMR for 2023. The first set of experiments includes using a univariate time series of AISMR values, and this set of experiments can be considered our baseline experiments since only previous AISMR values are used to predict the future AISMR value. The experiments were conducted by varying the value of lookback from the set {5, 10, 15, 20, 25}, and the mathematical formulation of these experiments can be done for any year *t*, given previous *k* years AISMR data, i.e., AISMR_*t*_, AISMR_*t*-1_, … AISMR_*t-k*+1_, predict the rainfall for the following year, i.e., AISMR_*t*+1_. The second set of experiments uses monthly Niño3.4 index values and the AISMR values. Multiple combinations of datasets were formed by varying the lookback for both AISMR and the Niño3.4 index. While AISMR was varied similarly to the category 1 experiment, i.e., from the set {5, 10, 15, 20, 25}, the lookback for the monthly Niño3.4 index was varied from 1 to 24 months. After evaluating the results using the two evaluation metrics RMSE percentage and Spearman correlation, lookback = 13 months for Niño3.4 index gave comparatively better results than other lookbacks. Hence, in further experiments, the previous 13 months of the Niño3.4 index were used along with varying lookbacks for AISMR. Thirteen months of the Niño3.4 index can be broken down into the first five months (January-May) of the year *t* + 1 and the last eight months (May-December) of the year *t* where *t* + 1 is the year for which we want to predict the AISMR value. The mathematical formulation of this set of experiments can be written as, at a particular year *t*, given the previous *k* years of AISMR data along with *m* months Niño3.4 index data before the JJAS season of the year *t* + 1, predict the rainfall for the following year, i.e., AISMR_*t*+1_. The final category of experiments incorporates the categorical IOD data along with AISMR and the Niño3.4 index. IOD informs the model whether the year for which we want to predict AISMR is a Positive, Neutral, or Negative IOD year. A one-hot encoding on the categorical IOD data gave three columns, each corresponding to a negative, neutral, and positive IOD year. For example, if the year is a positive IOD year, then the value of the three columns will be {0, 0, 1}, i.e., 0 for the negative and neutral columns, and 1 for the positive column. Mathematically formulating, for a year *t*, given the previous *k* years of AISMR data, 13 months of the Niño3.4 index, and categorical IOD data for the year *t* + 1, predict AISMR for the year *t* + 1. Similar to the last two categories of experiments, AISMR was varied for the lookback {5, 10, 15, 20, 25}. For each model trained, we find the *p*-value for a hypothesis test whose null hypothesis is that two sets of data are linearly uncorrelated. Using the *p*-values obtained from the models trained on each dataset, the average *p*-value and standard deviation were computed for every dataset type.

### Lookback period

For a given time step *t*, a lookback period of size *k* can be understood as the k previous time steps (including the current time step t) whose corresponding data will be given to the model to make the forecast for the time *t* + 1.

### RMSE percentage

RMSE percentage represents the root mean squared error between the actual and the predicted values in percentage terms with respect to the actual values. A lower RMSE percentage between the observations and forecasts implies better model performance.

$$RMSE\left(\text{\%}\right)=\sqrt{\frac{100}{n}{\sum }_{i=1}^{n}{\left(\frac{{Obs}_{i}-{Pred}_{i}}{{Obs}_{i}}\right)}^{2}}$$

### Spearman correlation

Spearman correlation represents the statistical measure of the strength of the relation between a ranked pair of variables. The Spearman correlation varies between  − 1 and  + 1, where positive correlation implies that if one variable increases, the other also increases and vice-versa. Similarly, a negative correlation implies if one variable decreases, the other variable increases and vice-versa. A correlation between the actual and predicted values closer to 1 signifies better model performance.

$$SpearmanCorrelation=1-\frac{6{\sum }_{i=1}^{n}{{d}_{i}}^{2}}{{n}^{3}-n}$$

*d*_*i*_–the difference between two rankings. *n–*the number of observations.

### Statistical significance test

In order to evaluate the statistical significance of the Spearman correlations, we find the *p*-value for a hypothesis test whose null hypothesis is that two sets of data are linearly uncorrelated. For each of the three datasets, the average and standard deviation of the *p*-values obtained for models trained for different lookback periods were computed.

### Input data of ENSO and IOD for the year 2023

The multi-model mean of the forecasts of Niño3.4 index provided by Columbia Climate School (https://iri.columbia.edu/our-expertise/climate/forecasts/enso/current/) and monthly IOD forecasts provided by the Bureau of Meteorology Australia (http://www.bom.gov.au/climate/enso/#tabs=Indian-Ocean) which indicated a positive IOD year have been used for making the AISMR forecast for the year 2023.

### Supplementary Information

Supplementary Information.

## Data Availability

All data used in this study are publicly available. The historical AISMR data used is obtained from Indian Meteorological Department (https://www.imdpune.gov.in/lrfindex.php). The Indian Ocean Dipole categorical data was obtained from Japan Meteorological Agency (https://ds.data.jma.go.jp/tcc/tcc/products/elnino/iodevents.html). The Nino3.4 index data used for training purpose is available at https://psl.noaa.gov/gcos_wgsp/Timeseries/Data/nino34.long.anom.data

## Code availability

All code used in this study is available from the corresponding author upon request.
