# Supplementary material for: Artificial intelligence predicts normal summer monsoon rainfall for India in 2023
Source: Sci Rep. 2024 Jan 17;14:1495. doi: 10.1038/s41598-023-44284-3 (PMC10794699; doi:10.1038/s41598-023-44284-3)
Supplement: Supplementary file 1 — Supplementary Information. [file 41598_2023_44284_MOESM1_ESM.docx]

**Supplementary Information**

|  |  | ***p*-value** | | |
| --- | --- | --- | --- | --- |
| **Model** | **Lookback** | **AISMR** | **AISMRNino** | **AISMRNinoIOD** |
| ARIMA | 5 | 0.2569 | - | - |
|  | 10 | 0.1562 | - | - |
|  | 15 | 0.1517 | - | - |
|  | 20 | 0.1044 | - | - |
|  | 25 | 0.4636 | - | - |
| LR | 5 | 0.2050 | 0.0130 | 0.0019 |
|  | 10 | 0.3275 | 0.0284 | 0.0062 |
|  | 15 | 0.3275 | 0.0320 | 0.0315 |
|  | 20 | 0.2862 | 0.0115 | 0.0076 |
|  | 25 | 0.1459 | 0.0105 | 0.0037 |
| SVR | 5 | 0.0050 | 0.0161 | 0.0070 |
|  | 10 | 0.1191 | 0.0058 | 0.0016 |
|  | 15 | 0.3161 | 0.0181 | 0.0103 |
|  | 20 | 0.2115 | 0.0093 | 0.0093 |
|  | 25 | 0.0152 | 0.0043 | 0.0043 |
| XGBoost | 5 | 0.1376 | 0.3218 | 0.2158 |
|  | 10 | 0.7286 | 0.0275 | 0.0167 |
|  | 15 | 0.3275 | 0.0117 | 0.0117 |
|  | 20 | 0.0974 | 0.0900 | 0.1051 |
|  | 25 | 0.0765 | 0.0155 | 0.0155 |
| LSTM | 5 | 0.1251 | 0.0014 | 0.0003 |
|  | 10 | 0.2115 | 0.0025 | 0.0031 |
|  | 15 | 0.5330 | 0.0058 | 0.0045 |
|  | 20 | 0.1328 | 0.0337 | 0.0252 |
|  | 25 | 0.0472 | 0.0167 | 0.0110 |
| CNN | 5 | 0.1864 | 0.0704 | 0.0755 |
|  | 10 | 0.1476 | 0.0167 | 0.0047 |
|  | 15 | 0.0865 | 0.0008 | 0.0067 |
|  | 20 | 0.0888 | 0.0076 | 0.0177 |
|  | 25 | 0.0819 | 0.0110 | 0.0210 |
| **Mean** | | **0.2033** | **0.0313** | **0.0247** |
| **Std dev** | | **0.1590** | **0.0640** | **0.0464** |

**Supplementary Table 1.** The p-values of the Spearman correlations obtained for each category of experiments using different data driven models. The mean and the standard deviation of each category is also noted.


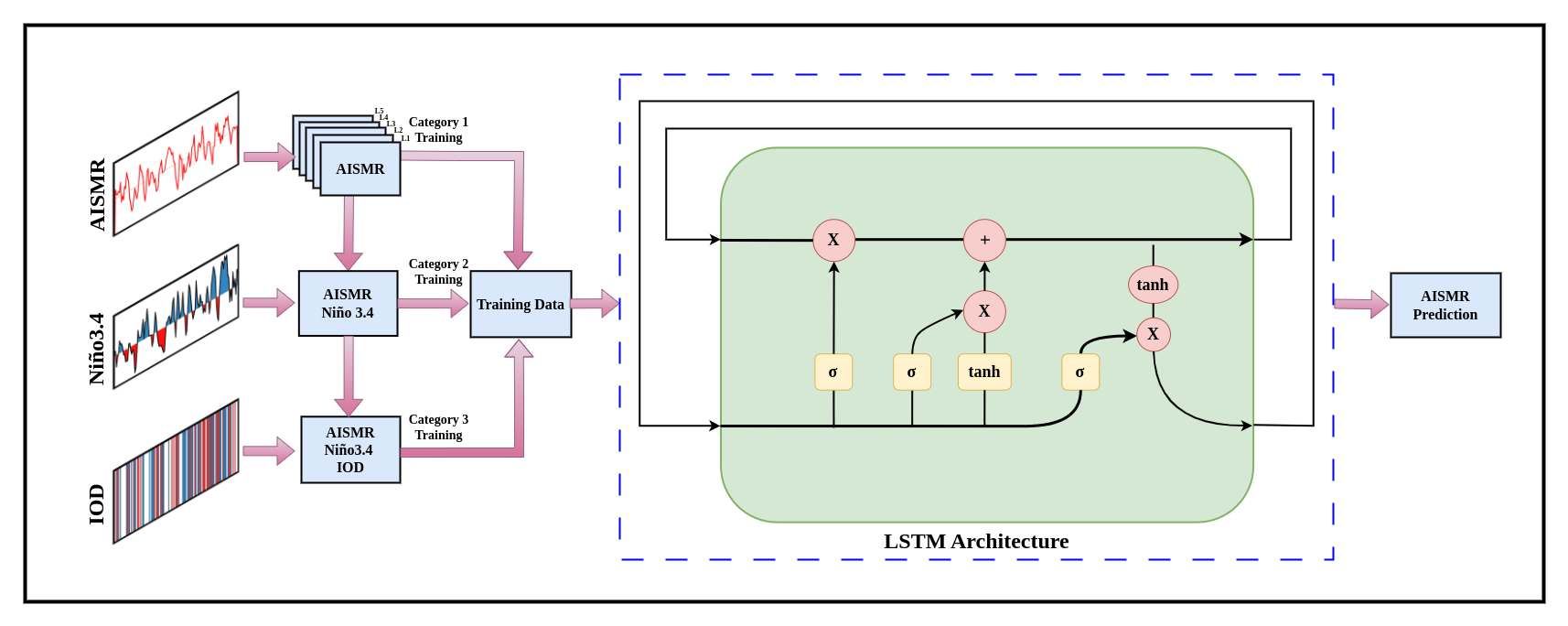


**Supplementary Figure 1. Schematic illustrating the different experiments along with Long Short-Term Memory (LSTM) architecture.** The figure provides a visual representation of the flow and processing of data. Category 1 training pathway represents training the model with only AISMR data, successively category 2 and 3 represents AISMR with Niño3.4 index and ASIMR with Niño3.4 index and IOD index respectively. L1, L2, L3, L4 stand in for the various AISMR lookback intervals that were utilized to train the model. Figure also pictorially represents the LSTM architecture, which is the best performing model used in AISMR prediction for the year 2023.
